# Emergence of HIV-1 drug resistance in antiretroviral therapy experienced perinatally infected children in South India

**DOI:** 10.1186/1471-2334-12-S1-P67

**Published:** 2012-05-04

**Authors:** Ujjwal Neogi, Pravat Nalini Sahoo, Ayesha De Costa, Anita Shet

**Affiliations:** 1 St. John’s National Academy of Health Sciences, Bangalore, India; 2 Karolinska Institute, Stockholm, Sweden

## Background

Over 25,000 HIV-infected children are currently alive and on highly active antiretroviral therapy (HAART) in India. Limited data are available on the efficacy of treatment and emergence of drug resistance (DR) in pediatric populations. In this study we aimed to characterize the pattern of drug resistance mutations (DRM) in a cohort of perinatally infected children.

## Method

Blood samples were collected from 61 children who were on first line of therapy for ≥ 6 months. Patients’ demographic and clinical parameters were documented. Viral load was measured using Abbott m2000rt system, Germany. DR Genotyping (using an in-house method) was performed on those with viral load >1000 copies/ml.

## Results

Among those who had been on ART for a median period of 24 months 51 children (83.6%) achieved virological suppression. There were 10 children with virological failure, and only two amongst these manifested immunological failure. Nine children (90%) had reverse transcriptase-related DRMs, and none had protease inhibitor-related DRMs. The most frequent NRTI mutation was M184V (n=9) followed by two thymidine analogous associated mutations (TAMs) M41L and T215Y (n=2). The most frequent nNRTI mutations were K103N (n=6) and Y181C (n=3).

## Conclusion

Our study showed a high proportion of children achieving virological suppression with a mean duration of two years of ART. Children with virological failure and drug resistance and without immunological failure will likely promote DRM accumulation and may jeopardise second-line ART options. These data suggest that virological monitoring may help optimise regimen switch in children.

